# Overexpression of Cotton *RAV1* Gene in *Arabidopsis* Confers Transgenic Plants High Salinity and Drought Sensitivity

**DOI:** 10.1371/journal.pone.0118056

**Published:** 2015-02-24

**Authors:** Xiao-Jie Li, Mo Li, Ying Zhou, Shan Hu, Rong Hu, Yun Chen, Xue-Bao Li

**Affiliations:** Hubei Key Laboratory of Genetic Regulation and Integrative Biology, School of Life Sciences, Central China Normal University, Wuhan, 430079, China; Wuhan University, CHINA

## Abstract

RAV (related to ABI3/VP1) protein containing an AP2 domain in the N-terminal region and a B3 domain in the C-terminal region, which belongs to AP2 transcription factor family, is unique in higher plants. In this study, a gene (*GhRAV1*) encoding a RAV protein of 357 amino acids was identified in cotton (*Gossypium hirsutum*). Transient expression analysis of the *eGFP:GhRAV1* fusion genes in tobacco (*Nicotiana tabacum*) epidermal cells revealed that *GhRAV1* protein was localized in the cell nucleus. Quantitative RT-PCR analysis indicated that expression of *GhRAV1* in cotton is induced by abscisic acid (ABA), NaCl and polyethylene glycol (PEG). Overexpression of *GhRAV1* in *Arabidopsis* resulted in plant sensitive to ABA, NaCl and PEG. With abscisic acid (ABA) treatment, seed germination and green seedling rates of the *GhRAV1* transgenic plants were remarkably lower than those of wild type. In the presence of NaCl, the seed germination and seedling growth of the *GhRAV1* transgenic lines were inhibited greater than those of wild type. And chlorophyll content and maximum photochemical efficiency of the transgenic plants were significantly lower than those of wild type. Under drought stress, the *GhRAV1* transgenic plants displayed more severe wilting than wild type. Furthermore, expressions of the stress-related genes were altered in the *GhRAV1* transgenic *Arabidopsis* plants under high salinity and drought stresses. Collectively, our data suggested that *GhRAV1* may be involved in response to high salinity and drought stresses through regulating expressions of the stress-related genes during cotton development.

## Introduction

Transcription factors play central roles in regulating the expression of downstream genes through trans-activating or trans-repressing elements binding to cis-acting elements in the promoters of target genes. Some characteristics (including DNA-binding specificity, transcriptional activation or repression, nuclear localization, interaction with other transcription factors or cofactors, and post-translational modifications) of the transcription factor are significant in the context of the ability to control target gene expression [1–3]. Up to now, an increasing number of transcription factors have been identified from higher plants, but the majority of them remain to be characterized in detail. It has been reported that the transcription factors of AP2, WRKY, bZIP, and MYB families in plants are involved in regulating the expression of defense genes in responses to biotic and abiotic stresses [4,5].

AP2 transcription factor family is particularly found in higher plants, and is divided into three subfamilies: AP2 (with two AP2 DNA-binding domains), ERF/DREB (with only one AP2 DNA-binding domain) and RAV (with an AP2 DNA-binding domain and a B3 domain) [4,6]. The AP2 and B3 DNA-binding domains differing in their biological functions are involved in distinct types of the transcription factors. In addition, no plant transcription factor has yet been demonstrated to contain two or more DNA-binding domains of distinct types, except for *Arabidopsis* RAV1 and RAV2 [7]. Although the members of AP2 subfamily are considered to be related to flower development, and ERF/DREB proteins participate in plant response to biotic and abiotic stresses, the function of RAV transcription factors are little elaborated so far.

RAV (related to ABI3/VP1) protein, which contains an AP2 domain in the N-terminal region and a B3 domain in the C-terminal region, is unique in higher plants [7]. Previous studies announced that the AP2 and B3 domains of RAV1 were proved to bind to CAACA and CACCTG motifs by binding site selection assays. In initial, the RAV genes, namely *RAV1* and *RAV2*, were identified in *Arabidopsis*, based on the sequence information of maize homologous gene *VIVIPAROUS1* (*Vp1*) [8]. An early study supposed that RAV1 protein may be as a negative regulator during *Arabidopsis* growth and development [9]. However, the later studies revealed that RAV1 positively regulates leaf maturation and senescence [10], controls the flowering time under long-day growth conditions [11], and participates in cold response [12]. Moreover, *RAV1* and *RAV2* genes display biphasic expression patterns in *Arabidopsis* response to internal and external stimulations [13]. Nowadays, more and more researches focus on the function of RAV proteins in plants. For example, a study indicated that tomato (*Solanum lycopersicum*) RAV enhances plant tolerance to the bacterial wilt [14]. GmRAV as a negative regulator acts on both photosynthesis and growth in soybean (*Glycine max*) [15]. In *Capsicum annuum*, CaRAV1 increases plant tolerance to drought and salt stresses [16]. Although a recent study reported that expression of a *RAV* gene in cotton is affected by salt stress [17], little is known on the role of *RAV* genes in cotton in detail yet.

Cotton (*Gossypium hirsutum*) is one of the most important crops in the world for its natural textile fiber and cottonseed oil. Cotton growth and development are adversely affected by a lot of environmental stresses (such as drought, high salinity and low temperature, etc.) during summer and autumn. Therefore, investigating the molecular mechanism of stress adaptation and tolerance of this plant species are of fundamental importance for improving cotton yield. In this study, a gene (*GhRAV1*) encoding a RAV DNA-binding protein was identified in cotton (*Gossypium hirsutum*). The expression of *GhRAV1* gene was induced by NaCl, polyethylene glycol (PEG), abscisic acid (ABA), and ethylene. Overexpression of *GhRAV1* in *Arabidopsis* increased plant sensitivity to salt and drought stresses.

## Materials and Methods

### Plant materials and growth conditions

Cotton (*Gossypium hirsutum* cv. Coker312) seeds were surface-sterilized with 70% (v/v) ethanol for 1 min and 30% (v/v) H_2_O_2_ for 1 h, followed by washing with sterile water. The sterilized seeds were germinated on half-strength Murashige and Skoog (MS) medium (pH 5.8) under a 16 h light/8 h dark cycle at 28°C for 6 days. Roots, hypocotyls and cotyledons were collected from these seedlings. The other tissues (such as leaves, petals, anthers, ovules, and different stage fibers) were derived from cotton plants grown in the trial field located in the Central China Normal University.

To detect response of the genes to abiotic stress, cotton seedlings grew for 5 days on half-strength MS medium, and then transferred onto the same medium supplemented with 20% polyethylene glycol (PEG), 150 mM NaCl and 100 μM abscisic acid (ABA) for 1–3 h. Cotton materials were collected from the treated seedlings for further experiments, using the untreated cotton plants as controls.

*Arabidopsis* seeds were sterilized with 2.5% NaClO solution for 5–10 minutes, followed by washing with sterile water. The sterilized seeds were germinated on MS medium under a 16 h light/8 h dark cycle at 23°C for 6 days, and then the seedlings were transferred into the soil for growth to maturation.

### Isolation of *GhRAV1* cDNA

Over 4,000 cDNA clones were randomly selected from a cotton seedling cDNA library for sequencing. Among them, one cDNA clone encoding an AP2 domain protein (designated as GhRAV1) was identified for further characterization.

### Quantitative RT-PCR analysis

Total RNA was extracted from tissues of cotton and *Arabidopsis*, respectively, by Trizol kit (Invitrogen) according to the manufacturer’s protocol. First-strand cDNA was synthesized from 1 μg DNase-treated total RNA sample using oligo (dT) and Takara MLV-Reverse transcriptase. Real-time quantitative RT-PCR analysis of cotton gene expression was performed using the fluorescent intercalating dye SYBR-Green in a detection system (Opticon2; MJ Research, New Haven, Connecticut, USA) as the method described earlier [18]. The Ct (cycle threshold), defined as the PCR cycle at which a statistically significant increase of reporter fluorescence is first detected, is used as a measure for the starting copy numbers of the target gene. Relative quantity of the target *GhRAV1* expression levels was performed using the comparative Ct method. Cotton *GhUBI1* and *GhACTIN* genes were used as standard controls in the RT-PCR reactions, and the gene-specific primers are shown in Table 1.

**Table 1:** Gene-specific primer pairs used in RT-PCR analysis for gene expression in cotton and Arabidopsis.

Gene name	Primer sequence
*GhRAV1*	5’-ATGTTGGTCTAGCTGGTGGATG-3’
	5’-GCTTTCTTCAGCCTTTTGTCTTG-3’
*GhACTIN*	5’-CTGCTGGAATCCATGAAACTAC-3’
	5’-TTCCTGTGGACAATGGATGGAC-3’
*GhUBI1*	5’-GGGATGCAAATCTTCGTGAAAAC-3’
	5’-CTGAATCTTCGCTTTCACGTTATC-3’
*AtRD29A*	5’-TGAAAGGAGGAGGAGGAATGGTTGG-3’
	5’-ACAAAACACACATAAACATCCAAAGT-3’
*AtRD29B*	5’- CCAGATAGCGGAGGGGAAAGGACAT-3’
	5’-AAGTTCACAAACAGAGGCATCATAC-3’
*AtABI1*	5’-AGATGGCAAGGAAGCGGATT-3’
	5’-CAACCACCACCACACTTATG-3’
*AtRAB18*	5’-AGATGGCAAGGAAGCGGATT-3’
	5’-CTTCTTCTCGTGGTGCTCAC-3’
*AtKIN1*	5’-ACCAACAAGAATGCCTTCCAAGC-3’
	5’- TCCCAACAGTTAATTAGAAAAGG-3’
*AtCOR15a*	5’-GCAGATGGTGAGAAAGCGAAAGAC-3’
	5’-AAGAATGTGACGGTGACTGTGGA-3’
*AtERD10*	5’-GAGGAAGAAGACCTGTTGG-3’
	5’-CCACGACCGACCGACGT-3’
*AtERD15*	5’-TCAGCGAGGCTGGTGGATG-3’
	5’-TGAGAATGGCGATGGTATCAGGA-3’
*AtACT2*	5’- GAAATCACAGCACTTGCACC-3’
	5’- AAGCCTTTGATCTTGAGAGC-3’

The expression of *GhRAV1* gene in the transgenic *Arabidopsis* plants was analyzed by quantitative RT-PCR, using *Arabidopsis ACTIN2* gene (*AtACT2*) as a quantitative control and the gene-specific primers (Table 1). To assay the expression of stress-related genes in the transgenic *Arabidopsis* plants, quantitative RT-PCR analysis was performed with the RNA samples isolated from four-week-old seedlings under NaCl and drought treatments, using the untreated seedlings at same developmental stage as controls.

All RT-PCR reactions were performed in triplicates, along with three independent repetitions of the biological experiments. Mean of three biological experiments was calculated for estimating gene expression levels.

### Protein Sequence and Phylogenetic Analysis

Unless otherwise stated, nucleotide and amino acid sequences were analyzed using DNAstar software (DNAStar Inc, Madison, WI, USA). Identification of protein domains and significant sites was performed with Motifscan (http://myhits.isbsib.ch/ cgibin/motifscan). Signal P (www.cbs.dtu.dk/services/SignalP/) was used to determine the N-terminal signal sequence. Sequence alignment was performed with ClustalX program (http://bips.ustrasbg.fr/fr/Documentation/ClustalX/). To investigate the evolutionary relationship of GhRAV1 protein with other RAV proteins, a phylogenetic tree was constructed by MEGA 5.0 program [19].

### Subcellular localization

The coding sequence of eGFP (enhanced green fluorescent protein) gene was cloned into pBluescript II SK vector to form an intermediate construct pSK-eGFP. Subsequently, GhRAV1 ORF (open reading frame, without the stop codon) was amplified by PCR and then cloned into the pSK-eGFP vector at a position upstream of the eGFP gene. The primers used in the PCR are GhRAV1GFP P1 (5’-CTTCCCGGGATGGATGGAAGCAGCATAG-3’) and P2 (5’-CTTTCTAGACAAAGCATCAATTACCCTTTG-3’). Then, the constructed GhRAV1:eGFP fusion gene was cloned into pBI121 vector, replacing the GUS gene. Tobacco leaf epidermal cells were injected with the GhRAV1:eGFP construct by *Agrobacterium*-mediated DNA transfer as described previously [20]. The cells transient-expressing *GhRAV1*:*eGFP* gene under the control of CaMV 35S promoter were selected for detecting GFP fluorescence on a SP5 Meta confocal laser microscope (Leica, Germany) with a filter set of 488 nm for excitation and 506–538 nm for emission, after the injected tobacco leaves were cultured on MS medium for 72 h. SP5 software (Leica, Germany) was employed to record and process the digital images taken [21].

### Transactivation activity assay

To investigate the transcriptional activity of *GhRAV1*, the coding sequence of *GhRAV1* was amplified by PCR using the proofreading *Pfu* DNA polymerase and gene-specific primers, and was cloned into *Eco*RI and *Xho*I restriction sites of pGBKT7 (Biosciences Clontech, Palo Alto, CA, USA) which containing the GAL4 DNA binding domain to create the fusion construct of pGBKT7-GhRAV1. The construct was introduced into yeast strain AH109 and Y187, and two reporter genes ADE2 and lacZ were tested by streaking the yeast AH109 transformants on SD/-Trp/-Ade medium (SD minimal medium lacking Trp and Ade) (Clontech Inc., Palo Alto, CA,USA) and the flash-freezing filter assay of yeast Y187 transformants, respectively. The gene-specific primers are GhRAV1 P1: 5’-CTTGAATTCATGGATGGAAGCAGCATAG-3’, and P2: 5’-CTTCTCGAGTTACAAAGCATCAATTACCC-3’. The yeast cells harboring pGBKT7 vector were used as negative control, and yeast cells harboring pGBKT7–53 which encoded a fusion of GAL4 DNA-BD/murine p53 and pGADT7-RecT, which encoded a fusion of the GAL4 DNA-AD/SV40 large T-antigen, was used as positive control.

### Phenotypic analysis of transgenic *Arabidopsis* seedlings

The coding sequence of *GhRAV1* gene, amplified from its cDNA by PCR with the proofreading *Pfu* DNA polymerase, was cloned into pMD vector under the control of CaMV 35S promoter. Primers used as follows: GhRAV1 P1, 5’-CTTCTCgAgTTACAAAgCATCAATTACCC-3’ and P2, 5’-CTTGAGCTCATTCTGACACCTTTCCATG-3’. The construct was then transferred into *Arabidopsis* by the floral dip method. Positive transformants were selected on MS medium with 50 mg/L kanamycin, and transferred in soil for growth until maturation and seed set. *GhRAV1* overexpression transgenic lines (T1 generation) were named as *GhRAV1*-T1oe. Homozygous transgenic lines of T2 and T3 generations were used for phenotypic analysis.

To analyze the *GhRAV1* overexpression plants, *Arabidopsis thaliana* Columbia ecotype was used for this study. After three washes in distilled water, transgenic *Arabidopsis* seeds were sown on Petri plates containing MS medium with or without abscisic acid (ABA), NaCl and polyethylene glycol (PEG). After the seeds were stratified at 4°C for 3 d, the agar plates were placed vertically in a culture room with a photoperiod of 16h light/8h dark at 22°C. The physiological indexes (including germination rate, green seedling rate, chlorophyll content, proline content and the maximum photochemical efficiency) were measured (n = 60 to 100).

*Arabidopsis* seedlings of the transgenic lines and wild type were transplanted into soil for further growing to maturation. Three-week-old seedlings were used for drought and NaCl treatments, respectively. For high salinity stress, the transgenic plants and wild type (control) in pots were watered with the same volume 300 mM NaCl for twice in continuous 2 days, and then kept in the NaCl-contained soil for 7 days. The status of plant tolerance to high salinity (NaCl) was observed. For drought stress, the transgenic plants and wild type (control) in pots were not watered for 7 days and then watered again for recovering. The status of plant tolerance to drought was observed. The physiological indexes and expression of the stress-related genes were also measured.

### Measurement of chlorophyll and proline contents

Chlorophyll content was determined on a fresh-weight basis. Total chlorophyll from 0.1 g leaves of ten-day-old seedlings with or without NaCl treatment was extracted with 95% ethanol, and chlorophyll concentrations were calculated by the method described earlier [20] using spectrophotometer (TU-1901) and microplate reader (Bioteck, USA). Differences in chlorophyll content between wild type and the transgenic seedlings were evaluated using a P value generated by a one-sided t-test.

Proline content was determined when the growing plants present the phenotype resisting to drought. Approximately 0.1g of plant leaves was homogenized in 10 ml 3% aqueous sulfosalicylic acid, and the homogenate was filtered through two filter papers. 2 ml filtrate was reacted with 2 ml acid-ninhydrin and 2 ml glacial acetic acid in a test tube for 1 hour at 100°C, and then the reaction was terminated in an ice bath. The reaction mixture was extracted with 4 ml toluene. Finally, the chromophore containing toluene was aspirated from the aqueous phase and warmed to room temperature. The absorbance of the samples was read at 520 nm in a spectrophotometer, using toluene as a blank. The proline concentration was determined from a standard curve and calculated on a fresh weight basis [22].

### Assay of maximum photochemical efficiency

The rosette leaves of *Arabidopsis* plants were used in the experiments to investigate the maximum photochemical efficiency. First, the leaves at same growing status were put in dark for 15 minutes, and then the maximum photochemical efficiency (Fv/Fm) of light system (PS II) in the leaves was measured by a photochemical efficiency tester (PEA, Hansatech Instruments, King’s Lynn, UK). The formula for calculating maximum photochemical efficiency is Fv/Fm = (Fm—Fo)/ Fm (Fm, maximal fluorescence; Fo, initial fluorescence; Fv, variable fluorescence; Fv/Fm, PS II photochemistry efficiency) [23].

### Statistic analysis

All the assays were repeated three times along with three independent repetitions of the biological experiments unless otherwise stated. The statistic analysis (Student’s t-test) was performed in the above experiments. Mean values were shown from three independent experiments with three biological replicates, and bars represent standard errors in experiments. Statistically (very) significant difference (* p<0.05, ** p<0.01) between the samples and controls was indicated by Student’s t-test.

## Results

### Isolation and characterization of *GhRAV1*

**Fig 1:**
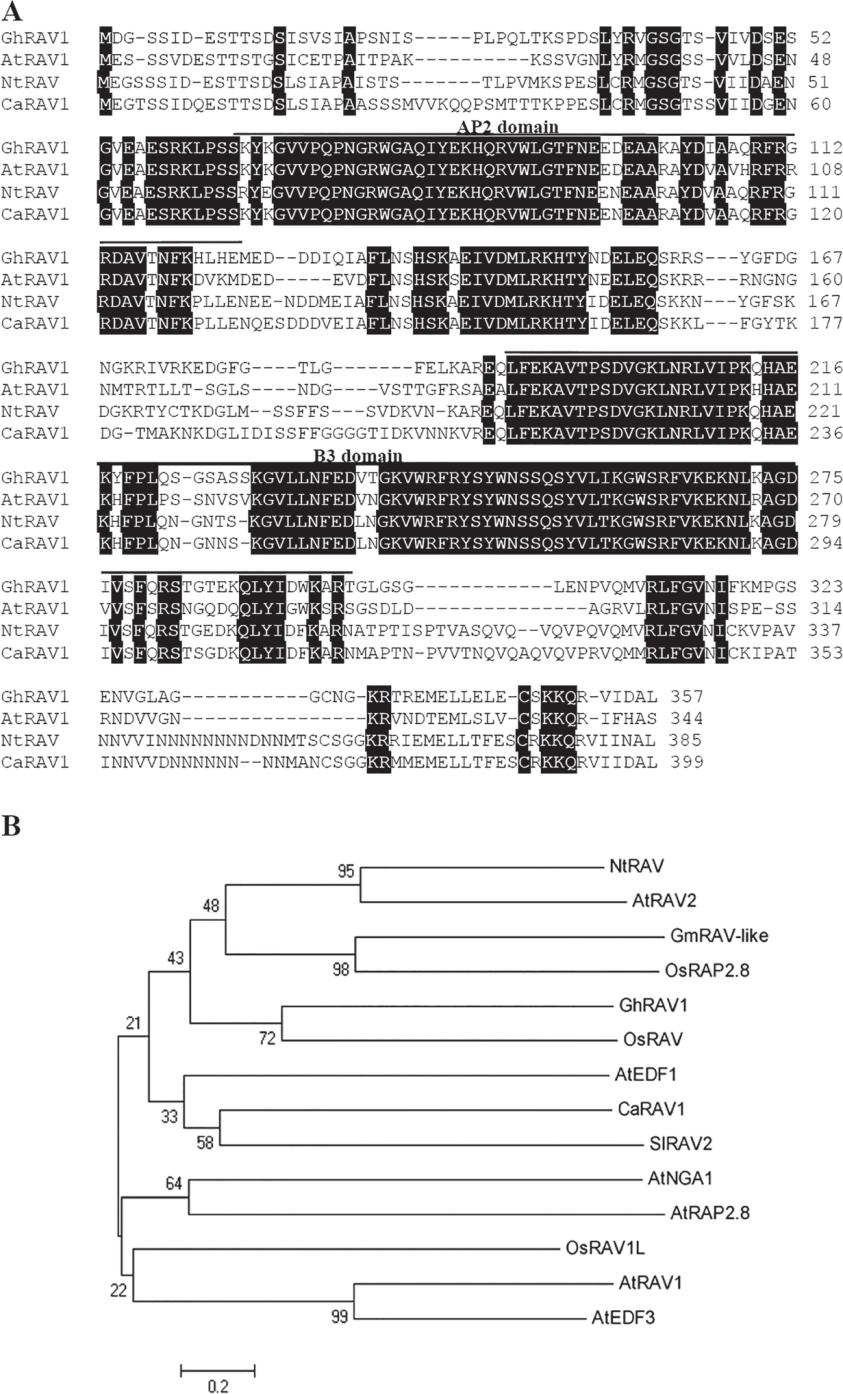
Characterization of GhRAV1. (**A**) Amino acid sequence alignment of GhRAV1 (*Gossypium hirsutum*), NtRAV (*Nicotiana tabacum*, EU870518), CaRAV1 (*Capsicum annuum*, AF478458) and AtRAV1 (*Arabidopsis thaliana*, NM_101197) by the ClustalX program. The conserved region is shadowed in black. AP2 and B3 domains are indicated by line. (**B**) Phylogenetic relationship of GhRAV1 with the other plant RAV proteins. GhRAV1 (*Gossypium hirsutum*) from this work, and the others from GenBank database. NtRAV (*Nicotiana tabacum*, EU870518); AtRAV1 (*Arabidopsis thaliana*, NM_101197); AtRAV2 (*Arabidopsis thaliana*, AY091069); OsRAP2.8 (*Oryza sativa Indica Group*, Q9AWS7); OsRAV (*Oryza sativa Japonica Group*, NM_001062772); OsRAV1L (*Oryza sativa Japonica Group*, NM_001050481); GmRAV-like (*Glycine max*, NM_001250671); *SlRAV2* (*Solanum lycopersicum*, EU164417); AtEDF3 (*Arabidopsis thaliana*, NM_113472); AtEDF1 (*Arabidopsis thaliana*, NM_102367); AtRAP2.8 (*Arabidopsis thaliana*, XM_002887162); AtNGA1 (*Arabidopsis thaliana*, NM_130254); CaRAV1 (*Capsicum annuum*, AF478458).

To investigate the roles of genes in cotton development and in response to abiotic stress, we randomly selected the thousands of cDNA clones from a cotton seedling cDNA library for sequencing. Among them, a full-length cDNA (designated as *GhRAV1*, accession number in GenBank: KJ801819) was identified to encode a RAV protein exhibiting significant homology with two distinct DNA binding domains of an AP2/B3-type transcription factor which is characteristic of the RAV DNA-binding proteins [7]. *GhRAV1* contains a 1074bp open reading frame encoding a protein of 357 amino acids. The predicted protein contains an AP2 domain in its N-terminal region. In addition, C-terminal region of GhRAV1 shows significant sequence similarity to the B3 domain of *Arabidopsis* ABI3 and maize VP1. Overall, the GhRAV1 protein shares high identity with *Arabidopsis* RAV1 (NP172784), tobacco (*Nicotiana tabacum*) RAV protein (NP172784), and *Capsicum annuum* RAV1 (AY727830) (Fig. 1A).

### Phylogenetic relationship of GhRAV1 with other RAV proteins

To determine divergence of the isolated GhRAV1 protein with the other plant RAV proteins during evolution, the phylogenetic relationship of 13 RAV domain proteins was analyzed by MEGA 5.0 program. As shown in Fig. 1B, these RAV proteins in the tree obviously split into two subgroups. GhRAV1 is located on a clade of the first subgroup, and shares high sequence homology with OsRAV, suggesting both proteins may have the same genetic evolution. It also shows relatively close relationship with NtRAV, AtRAV2, GmRAV-like and OsRAP2.8 on the sister lineage. On the other hand, AtNGA1, AtRAP2.8, OsRAV1L, AtRAV1 and AtEDF3 form the second subgroup.

### Expression of *GhRAV1* gene in cotton tissues under normal and stress conditions

**Fig 2:**
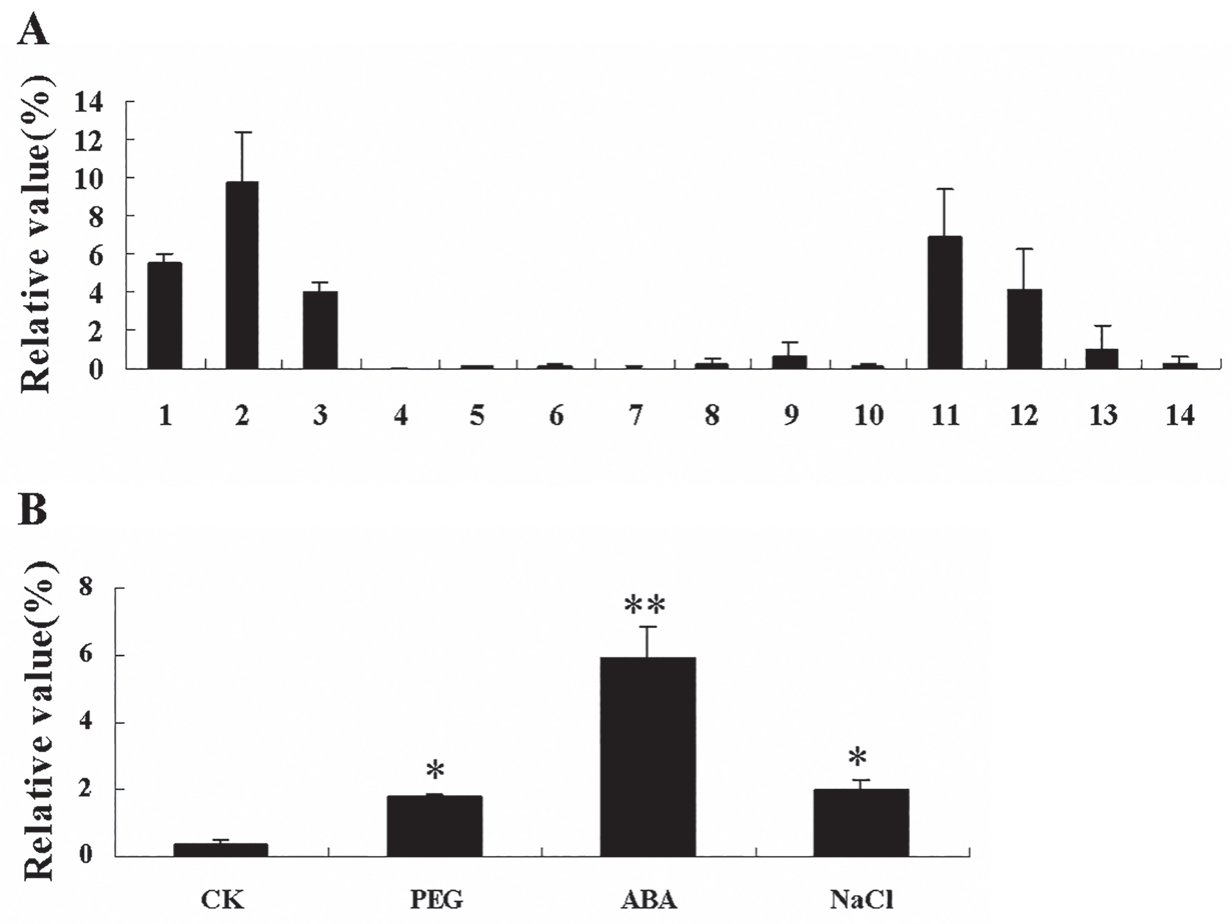
Quantitative RT-PCR analysis of GhRAV1 expression in cotton tissues and in cotton seedlings treated with NaCl, PEG and ABA. (**A**) Expression of *GhRAV1* in cotton tissues, including roots (1), cotyledons (2), leaves (3), petals (4), anthers (5), hypocotyls (6), ovules (7), and fibers at 3DAP (8), 6DAP (9), 9DAP (10), 12DAP (11), 15DAP (12), 18DAP (13) and 20DAP (14). (**B**) Expression of *GhRAV1* in five-day-old cotton seedlings under 100 μM ABA (abscisic acid), 150 mM NaCl and 20% PEG (polyethylene glycol) for 2h. Independent t-tests demonstrated that there was significant (*P < 0.05) or very significant (**P < 0.01) difference between the treated seedlings and controls. CK, cotton seedlings grew on MS medium without any treatment as controls (see Methods). DPA, day post anthesis.

*GhRAV1* expression was analyzed by quantitative RT-PCR. The experimental results showed that *GhRAV1* was strongly expressed in cotyledons, and at relative high levels in fibers, roots and leaves, but no or weak signals were detected in the other tissues. During early developmental stage (0–6 DPA), *Gh*RAV1 displayed its high expression in cotyledons. During fiber development, the highest expression of *Gh*RAV1 was detected in 12 DPA (day post anthesis) fibers, and then its expression was gradually declined to relatively low level (Fig. 2A).

The expression of *GhRAV1* gene was analyzed in cotton seedlings under abiotic stress conditions by quantitative RT-PCR. As shown in Fig. 2B, *GhRAV1* expression was dramatically induced after salt, PEG and ABA treatments. When cotton seedlings grew in normal conditions, very weak *GhRAV1* expression was detected in the seedlings. On the contrary, *GhRAV1* expression was strongly up-regulated in the seedlings treated with 150 mM NaCl, 20% PEG and 100 μM ABA, respectively, for 1–3 h, suggesting that *GhRAV1* may be involved in cotton response to high salinity and osmotic stress.

### GhRAV1 protein is localized in the cell nucleus

**Fig 3:**
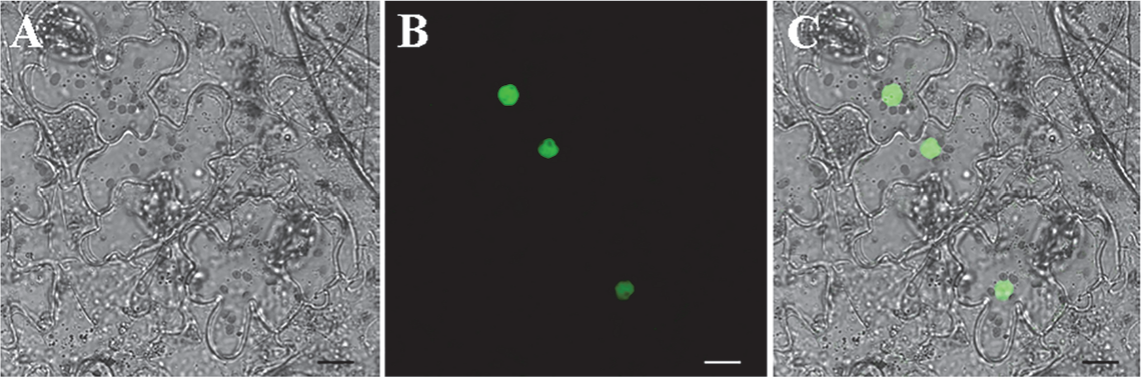
Subcellular localization of GhRAV1 protein in tobacco (*Nicotiana tabacum*) leaf epidermal cells. Fluorescence signals of GFP were mainly detected in nuclei of the leaf epidermal cells transiently expressing *GhGhRAV1*:*eGFP* fusion genes. (**A**) The bright field image. (**B**) Confocal microscopic image of GFP fluorescence. (**C**) Image B was merged with image A. Bar = 20 μm.

Bioinformatics analysis indicated that GhRAV1 protein contains no region that function as a nuclear localization signal. To investigate the subcellular localization of GhRAV1, green fluorescent protein (eGFP)-tagged GhRAV1 driven by cauli-flower mosaic virus (CaMV) 35S promoter was injected into tobacco (*Nicotiana tabacum*) leaf epidermal cells via *Agrobacterium*-mediated transformation. The transformed cells expressing GhRAV1:eGFP fusion proteins were examined with a Leica confocal laser scanning microscope. As shown in Fig. 3, strong GFP fluorescence was detected in cell nuclei of the tobacco leaf epidermal cells, suggesting that GhRAV1 protein is localized in the cell nucleus for its function in cotton.

### GhRAV1 protein lacks the activity of transcriptional activation

**Fig 4:**
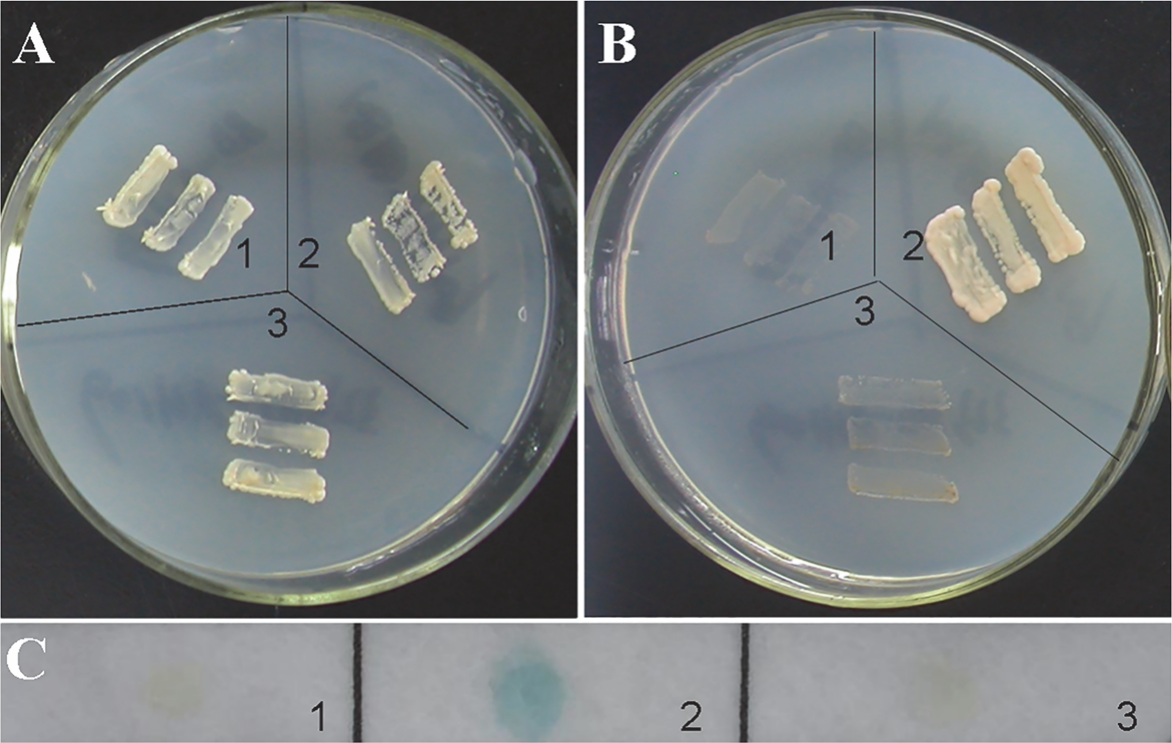
Transactivation activity assay of GhRAV1 protein in yeast cells. (**A**) Yeast AH109 transformants were streaked on SD/-Trp medium (SD minimal medium lacking Trp). (**B**) Yeast AH109 transformants were streaked on SD/-Trp/-Ade medium (SD minimal medium lacking Trp and Ade); (**C**) flash-freezing filter assay of the β-galactosidase activity in Yeast Y187 transformants. 1, Yeast transformants harboring pGBKT7 vector as negative control; 2, Yeast transformants harboring pGBKT7–53/pGADT7-RecT as positive control; 3, Yeast transformants harboring pGBKT7-GhRAV1 construct.

RAV transcription factors contain the conserved N-terminal DNA-binding domain (AP2) and C-terminal transcriptional repressor region, which can act as a repressor in regulatory pathways [16,24]. To assay the ability to activate transcription of HIS3 and ADE2 reporter genes from GAL4 upstream activation sequence, we transformed AH109 and Y187 yeast strain with pGBKT7-GhRAV1 construct. All the transformed yeast cells grew normally in SD medium lacking tryptophan (SD/–Trp) (Fig. 4A). However, the transformants with pGBKT7-GhRAV1 did not grow on the selective medium lacking tryptophan and adenine (SD/–Trp–Ade) (Fig. 4B). Furthermore, yeast cells expressing pGBKT7-GhRAV1 on the selective medium (SD/–Trp–Ade) did not turn blue at the presence of 5-bromo-4-chloro-3-indolyl-β-D-galactopyranoside (X-Gal), indicating that the reporter gene LacZ was not activated (Fig. 4C). These results suggest that GhRAV1 protein lacks the activity of transcriptional activation.

### Overexpression of GhRAV1 in *Arabidopsis* leads to the transgenic plants sensitive to ABA

**Fig 5:**
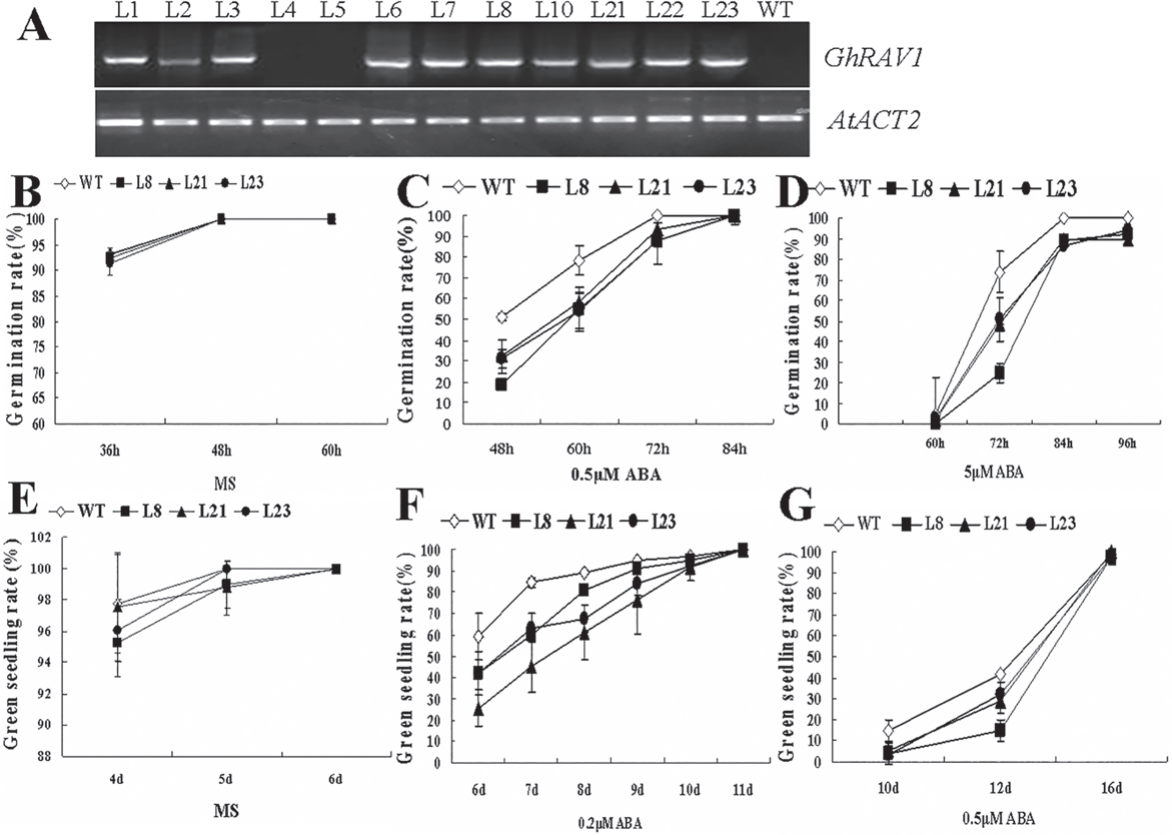
Assay of seed germination and seedling growth of the *GhRAV1*-overexpression transgenic *Arabidopsis* in the presence of exogenous ABA. (**A**) RT-PCR analysis of *GhRAV1* expression in the transgenic plants, using *Arabidopsis ACTIN2* gene (*AtACT2*) as a quantification control. (**B**) Statistic analysis of seed germination rate of the *GhRAV1* transgenic lines and wild type on MS medium (control). (**C**) Statistic analysis of seed germination rate of *GhRAV1* transgenic lines and wild type on MS medium with 0.5μM ABA. (**D**) Statistic analysis of seed germination rate of *GhRAV1* transgenic lines and wild type on MS medium with 5μM ABA. (**E**) Statistic analysis of green seedling rate of *GhRAV1* transgenic lines and wild type grown on MS medium (control). (**F**) Statistic analysis of green seedling rate of *GhRAV1* transgenic lines and wild type grown on MS medium with 0.2μM ABA. (**G**) Statistic analysis of green seedling rate of *GhRAV1* transgenic lines and wild type grown on MS medium with 0.5μM ABA. Independent t-tests demonstrated that there was significant difference (P < 0.05) in both seed germination and green seedling rates between the transgenic lines and wild type under ABA treatment (n > 200). WT, wild type; L1—L23, *GhRAV1*-overexpression transgenic lines 1–23.

To elucidate *GhRAV1* function in plants, we introduced *GhRAV1* gene into *Arabidopsis*, and obtained the transgenic plants overexpressing *GhRAV1* gene (Fig. 5A). Three homozygous *GhRAV1* transgenic lines (L8, L21 and L23) were selected for further analysis. Seeds of the transgenic lines and wild type germinated on MS medium containing various concentrations of abscisic acid (ABA), and the germination rates of both wild type and transgenic seeds were determined. The experimental results revealed that there was no significant difference in germination rates between the transgenic lines and wild type on MS medium without ABA (Fig. 5B). In the presence of exogenous ABA, the germination of both wild type and *GhRAV1* overexpression transgenic seeds was distinctly inhibited, but the degree of inhibition in the transgenic seeds was much greater than that of wild type. With 0.5 μM ABA treatment, nearly 60% of wild type seeds germinated for 48 hours, while only 16% to 35% of the transgenic seeds germinated at the same time. After 72 hours, the seed germination rate of the transgenic lines was less than 60%, but the wild type seeds still kept 80% of germination rate. The ultimate germination rate of *GhRAV1* transgenic seeds was significantly lower than that of wild type (Fig. 5C). The similar results were observed when treated the transgenic seeds and wild type seeds with 5μM ABA (Fig. 5D).

In addition, we determined the green seedling rate of both wild type and the transgenic lines. As shown in Fig. 5E, there was no significant difference in green seedling rate between the transgenic lines and wild type on MS medium (under normal conditions). In the presence of exogenous ABA, the green seedling rate of both wild type and *GhRAV1* overexpressing lines was significantly decreased. Under the treatment of 0.2 μM ABA for 7 days, nearly 85% of wild type seedlings become green, while about 50% green seedlings was observed in the transgenic lines (Fig. 5F). Similarly, with 0.5 μM ABA treatment, the green seedling rate of *GhRAV1* transgenic lines was also lower than that of wild type (Fig. 5G). The above results indicated that overexpression of *GhRAV1* in *Arabidopsis* enhances plant sensitivity to the exogenous ABA, suggesting that *GhRAV1* may participate in plant response to ABA signaling.

### Overexpression of *GhRAV1* in *Arabidopsis* results in the transgenic plants sensitive to high salinity

**Fig 6:**
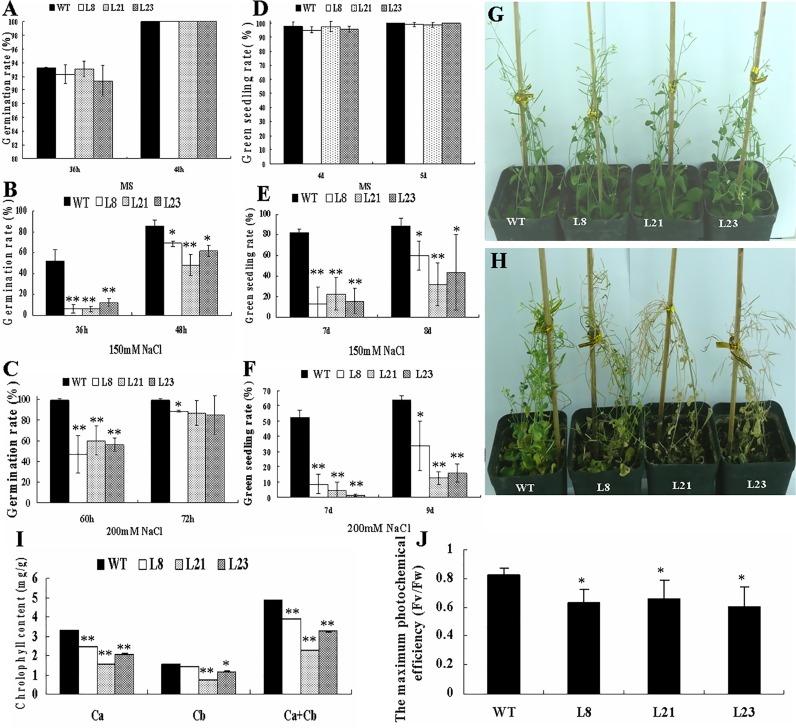
Phenotypic assay of the *GhRAV1*-overexpression transgenic *Arabidopsis* under high salinity stress. (**A**) Statistic analysis of seed germination rate of the *GhRAV1* transgenic lines and wild type on MS medium (control). (**B**) Statistic analysis of seed germination rate of *GhRAV1* transgenic lines and wild type on MS medium with 150mM NaCl. (**C**) Statistic analysis of seed germination rate of *GhRAV1* transgenic lines and wild type on MS medium with 200mM NaCl. (**D**) Statistic analysis of green seedling rate of *GhRAV1* transgenic lines and wild type grown on MS medium (control). (**E**) Statistic analysis of green seedling rate of *GhRAV1* transgenic lines and wild type grown on MS medium with 150mM NaCl. (**F**) Statistic analysis of green seedling rate of *GhRAV1* transgenic lines and wild type grown on MS medium with 200mM NaCl. (**G**) Phenotype of the *GhRAV1* transgenic plants and wild type grown in soil under normal conditions. (**H**) Phenotype of the *GhRAV1* transgenic plants and wild type grown in soil after watered with 150mM NaCl for one week. (**I**) Assay of chlorophyll content in leaves of the transgenic plants and wild type. 0.1g leaves were used to extract chlorophyll in a spectrophotometer (TU-1901) (see Methods). (**J**) Assay of maximum photosynthesis efficiency (Fv/Fw) in leaves of the transgenic plants and wild type. Mean values and standard errors were shown from three independent experiments with three biological replicates of plant materials. Independent t-tests demonstrated that there was (very) significant difference (* P < 0.05 or ** P < 0.01) between the transgenic lines and wild type under NaCl stress (n > 200). Ca, chlorophyll a; Cb, chlorophyll b. WT, wild type; L8, L21 and L23, three transgenic lines.

To investigate the role of *GhRAV1* in plant growth and development under high salinity, seeds of the transgenic lines and wild type germinated on MS medium without (control) or with various concentration of NaCl. As shown in Fig. 6A, there was no significant difference in seed germination between the transgenic lines and wild type under normal conditions (MS medium). In the presence of NaCl, the germination of both wild type and *GhRAV1* overexpression transgenic seeds was significantly inhibited, but the inhibition of transgenic seeds was greater than that of wild type. Under the treatment of 150 mM NaCl, over 60% of wild type seeds germinated, while only 10% of the transgenic seeds germinated after 36 hours (Fig. 6B). When treated with 200 mM NaCl, the germination rate of the *GhRAV1* transgenic seeds was significantly lower than that of wild type. Almost 100% of wild type seeds germinated, but only about 50% of the transgenic seeds germinated after 60 hours (Fig. 6C).

The green seedling rate of both wild type and the transgenic lines was also determined. The experimental results revealed that there was no significant difference in green seedling rate between the transgenic lines and wild type on MS medium without salt (Fig. 6D). In the presence of NaCl, the green seedling rate of both wild type and *GhRAV1* overexpression lines was significantly declined. However, the green seedling rate of the transgenic lines was much less than that of wild type. With treatment of 150mM NaCl, nearly 80% of wild type seedlings became green, but only about 10% green seedlings of the transgenic lines was observed after 7 days (Fig. 6E). When treated with 200mM NaCl, the green seedling rate of *GhRAV1* transgenic lines was also lower than that of wild type. Nearly 60% of wild type seedlings become green, but about 20% green seedlings of the transgenic lines were found after 9 days (Fig. 6F). These results indicated that overexpression of *GhRAV1* in *Arabidopsis* resulted in plant sensitive to high salinity.

Moreover, chlorophyll content and maximum photochemical efficiency of the transgenic plants were measured, using wild type as control. The results revealed that both chlorophyll content and maximum photochemical efficiency of two-week-old transgenic plants were lower than those of wild type. We used the high salt low permeability (as Methods) to evaluate the changing trend of chlorophyll content in the transgenic plants after NaCl treatment. The same growth-status *Arabidopsis* leaves were transferred onto MS medium supplemented with 150 mM NaCl for three days. The results indicated that leaves of the transgenic plants became yellow earlier than those of wild type. The 3-week-old *Arabidopsis* plants grown in pots were watered with 150 mM NaCl solution. After 7 days, the transgenic plants were getting to wilting earlier than wild type (Fig. 6G and 6H). Both chlorophyll content and maximum photochemical efficiency in the *GhRAV1* overexpression plants were lower than those in wild type (Fig. 6I and 6J). Collectively, the data suggested that *GhRAV1* may participate in plant response to high salinity, and make the transgenic plants sensitive to salt stress.

### Overexpression of *GhRAV1* in *Arabidopsis* enhances drought sensitivity of the transgenic plants

**Fig 7:**
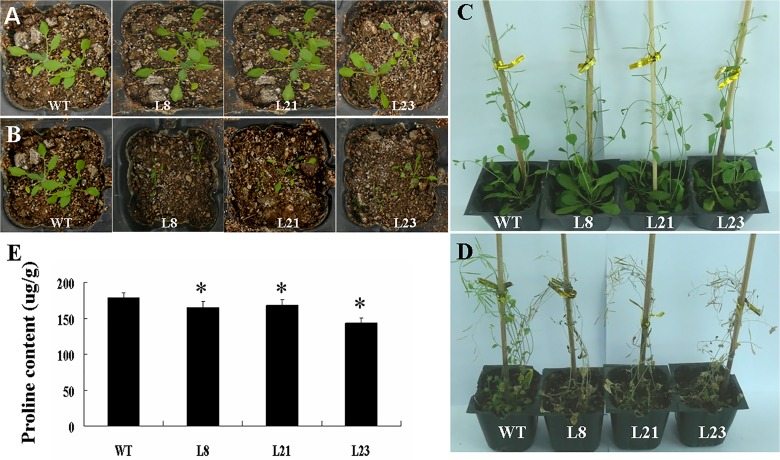
Phenotypic assay of the *GhRAV1*-overexpression transgenic *Arabidopsis* under drought stress. (**A**) Young *GhRAV1* transgenic seedlings and wild type grown in soil under normal conditions (control). (**B**) Young *GhRAV1* transgenic seedlings and wild type grown in soil under drought stress for one week. (**C**) *GhRAV1* transgenic plants and wild type grown in soil under normal conditions (control). (**D**) *GhRAV1* transgenic plants and wild type grown in soil under drought stress for one week. (**E**) Assay of proline content in leaves of the transgenic plants and wild type plant. Mean values and standard errors were shown from three independent experiments with three biological replicates of plant materials. Independent t-tests demonstrated that there was significant difference (* P < 0.05) in proline content between the transgenic lines and wild type under drought stress. WT, wild type; L8, L21 and L23, three transgenic lines.

Seeds of the *GhRAV1* overexpression transgenic lines and wild type grow one week in pots to do the drought-stress experiments. The soil in pots allowed drying for 7 days by withholding water. As shown in Fig. 7A, both young *GhRAV1* transgenic plants and wild type grew well in pots before drought treatment. However, the transgenic plants displayed severe wilting, whereas the wild type plants still grew normally under drought treatment (Fig. 7B). Subsequently, we chose two-week-old *Arabidopsis* plants grown in pots to do the drought-stress experiments. The soil in pots allowed drying for 7 days by withholding water. As shown in Fig. 7C, both *GhRAV1* transgenic plants and wild type grew well in pots before drought treatment. However, the transgenic plants displayed severe wilting, whereas the wild type plants still grew normally under drought stress (Fig. 7D). The proline content in transgenic plants was lower than that of wild type (Fig. 7E). These results suggested that overexpression of *GhRAV1* in Arabidopsis enhances the drought sensitivity of the transgenic plants.

### Expressions of ABA-, salt- and drought-related genes are altered in the transgenic *Arabidopsis*

**Fig 8:**
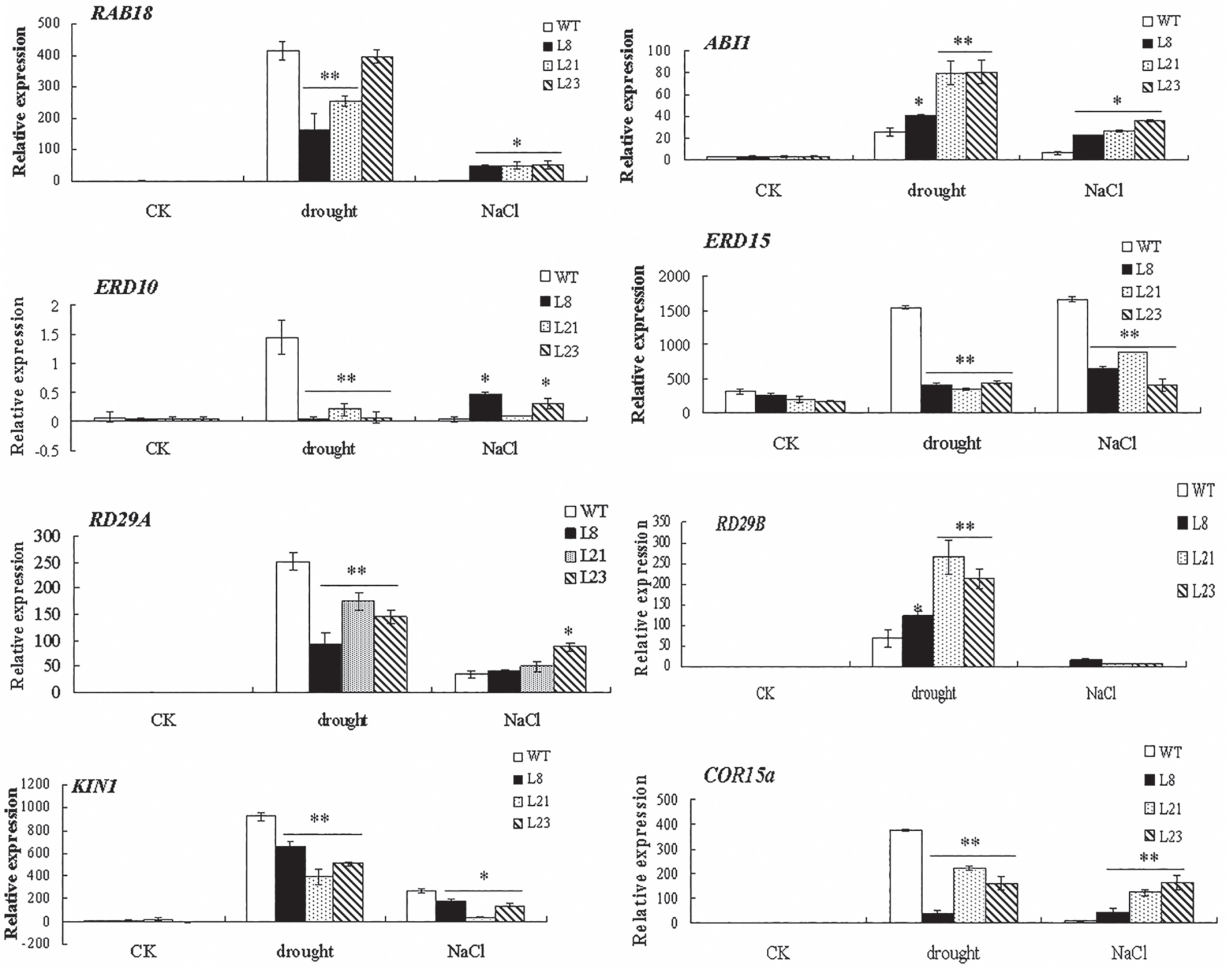
Quantitative RT-PCR analysis of expressions of the stress-related genes in *GhRAV1*-overexpression transgenic Arabidopsis under drought and salt stresses. Total RNA was isolated from the four-week-old transgenic plants and wild type grown under normal conditions and drought and NaCl stresses (see Methods). Transcript levels of *Arabidopsis RAB18, ABI1, ERD10, ERD15, KIN1, RD29A, RD29B* and *COR15a* genes in the transgenic plants and wild type were determined by quantitative RT-PCR, using *Arabidopsis ACTIN2* gene (*AtACT2*) as a quantification control. Mean values and standard errors were shown from three independent experiments with three biological replicates of plant materials. Independent t-tests demonstrated that there was significant difference (* P < 0.05;**P<0.01) in gene expression level between the transgenic lines and wild type. WT, wild type; L8, L21 and L23, three *GhRAV1* transgenic lines.

To investigate whether overexpression of *GhRAV1* in *Arabidopsis* affects the expressions of the genes involved in plant response to abiotic stress, we analyzed expression levels of some genes, including *RAB18* (response to ABA), *ABI1* (ABA-insensitive factor), *ERD10* and *ERD15* (early responsive to dehydration), *RD29A* and *RD29B* (responsive to desiccation, basic responsive gene in the stress), *COR15a* (cold-regulated) and *KIN1* (a plasma associated protein kinase that regulates the cell surface). As shown in Fig. 8, expressions of these marker genes were at relatively low levels in the transgenic plants and wild type grown in normal conditions. However, all the genes were strongly induced in the transgenic lines and wild type under NaCl and drought treatments. Furthermore, there were significant difference in expression level of the genes between the transgenic lines and wild type under drought stress. Compared with those in wild type, expressions of *RAB18, ERD10, ERD15, RD29A, KIN1* and *COR15a* were remarkably declined, while transcripts of *ABI1* and *RD29B* were increased in the transgenic plants under drought conditions. With 300 mM NaCl treatment, expression levels of *ERD15* and *KIN1* genes were significantly lower in the transgenic lines than those in wild type, but expressions of *RAB18, ABI1, ERD10, RD29A* and *COR15a* were more or less enhanced in the transgenic plants compared with those in wild type. These results suggested that *GhRAV1* may participate in response to salt and drought stresses by regulating the expressions of the stress-related genes during plant growth and development.

## Discussion

It has been known that RAV proteins containing one AP2 domain and one B3 domain belong to the AP2/EREBP family [25,26]. In this study, we identified a cotton RAV protein (GhRAV1) that has the conserved features of the AP2/EREBP protein family. Generally, plant transcription factors contain nuclear localization signals (NLS) that vary in sequence, organization and number [3]. Previous studies indicated that the pepper RAV1 (CARAV1), *Galegae orientalis* RAV (GoRAV) and *Arabidopsis* RAV1 (AtRAV1) proteins are localized in the cell nucleus [10,16,27]. Although bioinformatics analysis did not find the NLS in GhRAV1 sequence, our experimental results revealed that GhRAV1:GFP fusion proteins were localized in the nuclei of tobacco epidermal cells, suggesting GhRAV1 contains an unknown NLS in its sequence. In addition, it has been reported that some RAV proteins are transcription repressors in rice, pepper and *Arabidopsis* [16,24,28]. Similarly, we also found *GhRAV1* has no transcriptional activation in yeast cells, implying that *GhRAV1* might function as a negative regulatory component in regulating expression of its target genes during cotton development and in response to environmental stress.

Environmental stresses (such as cold, drought, high salinity and mechanical wounding, etc) induce the synthesis of abscisic acid (ABA) [29], and ABA plays a cardinal role in plant adaptation to stress [30]. Previous studies indicated that *AtRAV1, AtRAV2, CARAV1* and *SlRAV2* were strongly induced in plants after treated with bacterial pathogen, salicylic acid (SA), high salinity and mannitol [13,14,16,27,31]. In this study, our data revealed that the expression of *GhRAV1* in cotton seedlings is induced by NaCl, PEG and ABA, suggesting that *GhRAV1* may play a role in cotton response to abiotic stress and ABA signaling. Previous studies demonstrated that RAV proteins integrate into the defense-response pathway in plants [32,33]. To further understand the role of *GhRAV1* in plant response to stress, we generated the transgenic *Arabidopsis* plants overexpressing *GhRAV1*. Under salt, drought and ABA treatments, we found both seed germination and green seedling rates were declined in the transgenic lines, compared with those of wild type. This may indicate that *GhRAV1* may take part in plant response to salt/drought stress in an ABA-dependent manner.

High salinity can poison the cells and reduce the photosynthesis of plants [34]. Sohn and colleagues (2006) found *CARAV1* enhanced the transgenic plant resistance to NaCl, but sensitive to ABA [16]. On the contrary, our data in this study revealed that *GhRAV1* transgenic plants were sensitive to both high salinity and exogenous ABA. In addition, a study reported that *AtRAV1* positively regulates leaf senescence, leading to earlier leaf etiolate [10]. In soybean, a RAV-like transcription factor controls photosynthesis and senescence [15]. In our study, *GhRAV1* transgenic plants display the high salinity and drought sensitivity. Under salt and drought stresses, consequently, the transgenic plants present lower chlorophyll content and earlier get into etiolate phenomenon than those of wild type.

The abiotic stress-related genes (such as *RD29A, RD29B, RAB18, ABI1, ERD15, KIN, ERD10* and *COR15a* etc.) have been widely used as markers in studying plant response and defense to environmental stress. Previous studies indicated that these genes in plants are often responsive to osmotic, low-temperature, drought or/and high salinity stresses [35–38]. The altering expression of these marker genes may be helpful for plant resisting to the abiotic stress [13,14,16,27,31]. For example, ABI1 (ABA-insensitive1) is a negative regulator in plant response to ABA [37]. KIN1 is important for protecting the components of the cell, and is up-regulated in plants under high salinity treatment [39]. ERD10 belong to the dehydrin (DHN) family, also known as group 2 LEA proteins in *Arabidopsis* [40]. Expression of *ERD10* is up-regulated in plants response and tolerance to low temperature, high salinity and drought via the abscisic acid cascade [41]. Moreover, *RD29A* gene participates in response to osmotic and cold stresses, mediated by both ABA-dependent and ABA-independent pathways [42]. COR15a appears to function by decreasing the tendency of membranes to form the lamella-to-hexagonal II phase, which leads to membrane damage during freezing [43]. In this study, our data indicated that expressions of some stress-related genes were altered in *GhRAV1*-overexpression transgenic plants under drought and salt stresses, suggesting that *GhRAV1* may participate in plant response to abiotic stress through regulating expressions of the stress-related genes during plant development.
